# High Effectiveness of the Changchun Baike Varicella Vaccine in a Real-World Outbreak Setting: An Observational Study from Yanji City, China

**DOI:** 10.3390/vaccines14010042

**Published:** 2025-12-30

**Authors:** Zheng Wang, Shuhan Shang, Xiaoguang Guo, Shiyuan Song, Feng Guo, Na Xu, Feifan Ren, Zijian Chen, Yihua Li, Hanxue Gu

**Affiliations:** 1 Department of Preventive Medicine, Medical School, Yanbian University, Yanji 133000, China13789724993@163.com (S.S.); 15370075832@163.com (F.R.); 19843619325@163.com (Z.C.); 2 Medical College, Yanbian University, Yanji 133000, China; 3 Market Medicine Department, Changchun Baike Biotechnology Co., Ltd., Changchun 130012, China; 4 Department of Health Economy and Security Research, China National Health Development Research Center, Beijing 100000, China; 5 Clinical Department, Changchun Baike Biotechnology Co., Ltd., Changchun 130012, China

**Keywords:** varicella, live attenuated varicella vaccine, epidemiological characteristics, protective effect

## Abstract

**Objectives**: This study aimed to investigate the protective effect of the Changchun Baike varicella vaccine in Yanji City from 2018 to 2024. **Methods**: Varicella surveillance data from 2018 to 2024 and vaccination records from 2018 to 2020 were collected from the China Disease Prevention and Control Information System and analyzed. **Results**: In total, 2452 varicella cases were reported in Yanji from 2018 to 2024, with an average annual incidence rate of 62.71 per 100,000 population. Notably, the annual incidence rate decreased from 142.37 per 100,000 in 2018 to 55.25 per 100,000 population in 2024. Additionally, the highest and lowest incidence rates were observed in the 10–14 and ≥40 years age groups, respectively. Moreover, the vaccine demonstrated high protective effectiveness of 98.0–99.0% for one dose and 99.0% for two doses across the study period. These estimates were derived from propensity score-matched cohorts ranging from 686 to 6990 individuals (343 to 3495 matched pairs) across three overlapping observation periods (2018–2022, 2019–2023, and 2020–2024). **Conclusions**: The two-dose varicella vaccination schedule demonstrated superior protective efficacy compared with the single-dose schedule.

## 1. Introduction

Varicella, caused by the varicella zoster virus (VZV), is highly contagious and primarily transmitted through respiratory droplets or direct contact [1]. Varicella affects individuals of all ages, with a higher incidence among infants, preschool, and school-aged children, often leading to outbreaks [2]. Globally, approximately 140 million people are infected with varicella annually, posing a significant public health challenge [3]. Recently, varicella has become the leading vaccine-preventable infectious disease among children in China, with a national incidence rate of approximately 25–30 per 100,000 population in 2022, highlighting the urgent need for enhanced control measures [4,5]. As an economic and transportation hub in Jilin Province with a dense population, Yanji City is prone to varicella outbreaks in schools and childcare facilities. Vaccination is the most effective method for preventing the disease [6]. The Changchun Baike varicella vaccine, a live attenuated vaccine based on the Oka strain, has been licensed and widely used in China since 2013. It shares the same viral strain as international vaccines such as Varivax and Varilrix, with comparable immunogenicity demonstrated in Chinese populations. In China, the recommended vaccination schedule consists of two doses: the first at 12–24 months of age and the second at 4–6 years of age, with some regions offering free two-dose programs. Notably, the Changchun Baike vaccine is the primary vaccine used in Yanji’s immunization program, contributing to a reduction in the average annual incidence rate to 62.71 per 100,000 population between 2018 and 2024. However, single-dose vaccination has limited protective efficacy, and breakthrough infections may occur [7]. In this study, we conducted an epidemiological analysis of varicella in Yanji from 2018 to 2024 and evaluated the protective efficacy of varicella vaccine from 2018 to 2020 to provide guidance for varicella prevention and control.

## 2. Materials and Methods

### 2.1. Case Definition and Diagnostic Criteria

Varicella cases were reported by clinicians and confirmed according to the national diagnostic guidelines of China (Diagnostic Criteria for Varicella, WS 274-2007). Cases were classified as clinically confirmed based on a typical maculopapular vesicular rash with or without fever [8]. Laboratory confirmation (PCR or serology) was not routinely performed and was conducted only in a minority of severe or atypical cases. To maximize specificity, the primary analysis included only clinically confirmed cases; suspected cases were excluded. No substantial changes occurred in diagnostic or reporting criteria during 2018–2024 that would systematically affect case ascertainment. Breakthrough varicella cases were defined as rash onset >42 days after vaccination [8].

### 2.2. Data Sources

Varicella case data were obtained from the China Disease Prevention and Control Information System and the Infectious Disease Reporting System. Varicella cases were filtered for residents of Yanji with onset dates of 1 January 2018, to 31 December 2024, totaling 2452 confirmed cases, excluding suspected cases. Additionally, varicella vaccination data from 2018 to 2020 were collected for this study.

### 2.3. Data Quality and Completeness

Vaccination records encompassed both public and private clinics registered in the national immunization information system. Population denominators were derived from official annual reports of the Yanji Municipal Bureau of Statistics. Individuals with missing or unknown vaccination status were excluded from the vaccine effectiveness analysis. Data quality was maintained through routine cross-verification by local Centers for Disease Control and Prevention (CDC) staff. Annual varicella incidence rates were calculated as the number of reported confirmed cases in a given year divided by the mid-year population estimate for Yanji City, multiplied by 100,000, using the following formula:

Incidence rate (per 100,000 population) = (Number of confirmed varicella cases/Mid-year population) × 100,000.

### 2.4. Methods

Descriptive epidemiological methods were used to analyze the temporal, spatial, and population distributions of varicella incidence in Yanji from 2018 to 2024. Additionally, a screening method was employed to assess varicella vaccination rates and protective efficacy. Individuals were divided into vaccinated and unvaccinated groups based on vaccination status in 2018, 2019, and 2020, categorized as one dose, two doses, or both. Disease occurrence was observed from 2018 to 2022, 2019 to 2023, and 2020 to 2024. Cases were matched by age (<15 years), excluding those with onset within 1 year of birth or within 42 days post-vaccination. Matched cases were included in the protective efficacy analysis of the Changchun Baike varicella vaccine.

Due to imbalances in variables, such as age and dose number between the vaccinated and unvaccinated groups, propensity score matching (PSM) was used to balance covariates. A 1:1 matching ratio was applied with a caliper value of 0.2 and random seed of 50. Logistic regression was used to estimate propensity scores, and nearest-neighbor matching was used. Considering that the vaccinated group was smaller, the unvaccinated group was matched with replacement, with each unvaccinated subject matched no more than three times [9].

### 2.5. Statistical Analysis

Data collected in this study were organized using Excel 365. Descriptive epidemiological methods and SPSS 22.0 were used for count data analysis, with chi-square tests (*p* < 0.05 indicating statistical significance). Varicella incidence trend was analyzed using the Joinpoint regression model (Joinpoint Regression Program 4.8.0.1). Annual percent change (APC), the primary trend indicator generated by the Joinpoint regression model, was used to quantify the average percentage change in varicella incidence rates per year. Data were processed using SPSS 24.0 after PSM was performed using the MatchIt package in R (version 4.4.1). Additionally, the survival package was used for stratified Cox regression analysis. Covariate distributions between the vaccinated and unvaccinated groups were compared using chi-square tests (α = 0.05). Notably, the Kaplan–Meier survival curves showed no crossover, meeting the Cox regression assumptions. Disease occurrence was the dependent variable, with covariates included in the stratified Cox regression model to calculate the adjusted hazard ratios (aHR) and 95% confidence intervals (CI). Additionally, adjusted vaccine efficacy (aVE) was calculated as (1 − aHR) × 100% [9].

### 2.6. Ethical Reflections

This study was approved by the Institutional Review Board of Yanbian University (Ethics Code: 10249). The research was conducted in accordance with the principles of the Declaration of Helsinki.

The study utilized anonymized surveillance data from the national China Disease Prevention and Control Information System and local CDC, as well as aggregated vaccination records collected from community vaccination units. All data were fully de-identified prior to analysis; no personal identifiers (e.g., names, ID numbers, or precise addresses) were accessed or retained. Vaccination records were aggregated at the community level before integration with surveillance data. Data handling, transfer, and storage strictly complied with national regulations on infectious disease reporting and personal information protection.

As this was a retrospective analysis of routinely collected public health surveillance and immunization program data, the Institutional Review Board waived the requirement for individual informed consent. Blank informed consent templates were submitted during the ethical review process as required by the committee.

## 3. Results

### 3.1. Temporal Distribution

**Figure 1:**
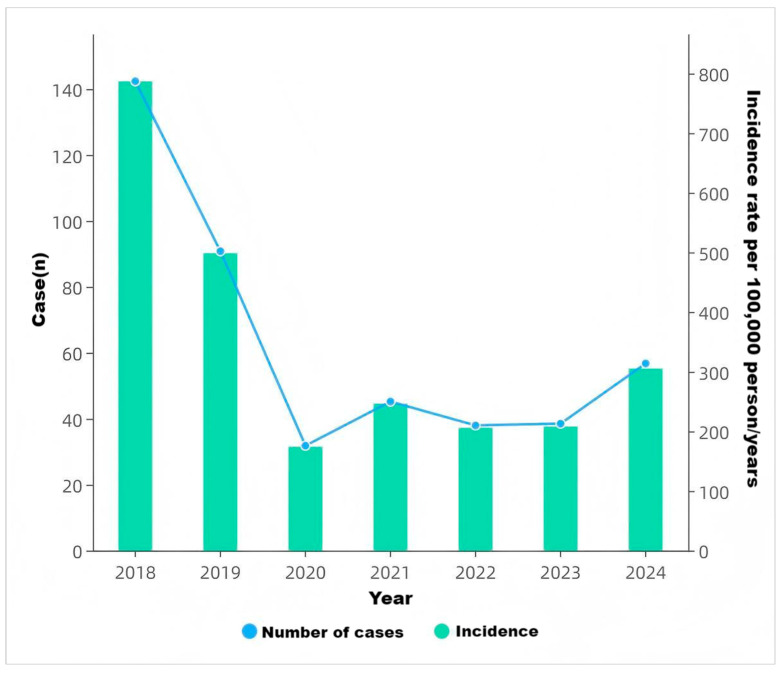
Varicella case counts and incidence rates in Yanji city from 2018 to 2024.

**Figure 2:**
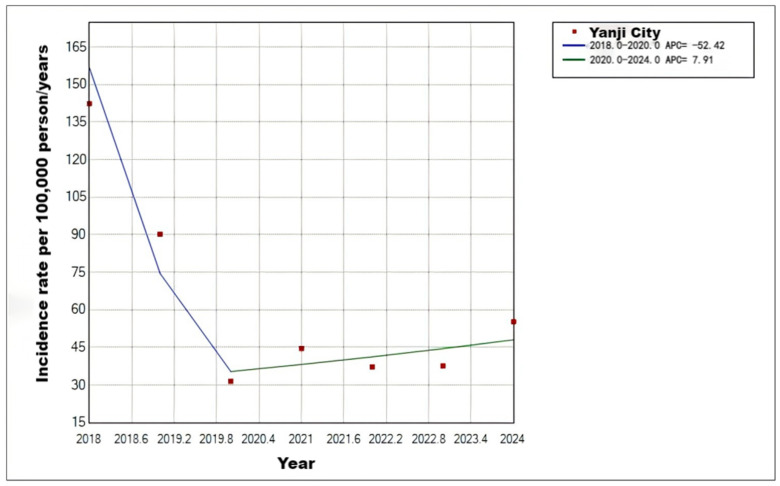
Trends in varicella incidence rates in Yanji city from 2018 to 2024.

**Figure 3:**
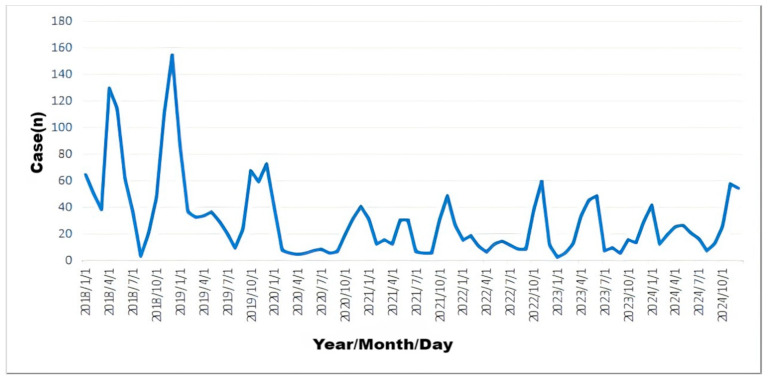
Monthly distribution of varicella cases in Yanji city from 2018 to 2024.

In total, 2452 varicella cases were reported in Yanji from 2018 to 2024, with an average annual incidence rate of 62.71 per 100,000 population. Although not statistically significant, the incidence rate decreased during this period. Cases were reported monthly, with a biphasic pattern peaking from April to July (31.83%, 2219/6972 cases) and from October to January of the following year (51.71%, 3605/6972 cases) (Figure 1, Figure 2 and Figure 3).

### 3.2. Spatial Distribution

**Figure 4:**
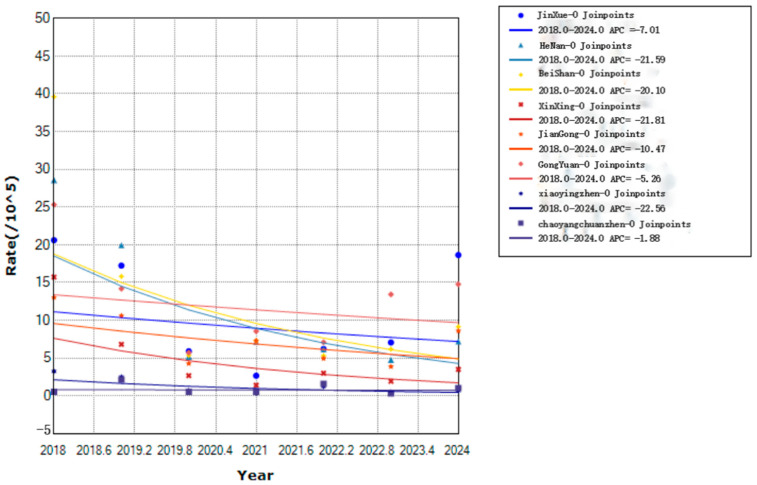
Trends in varicella incidence rates in six streets and four towns in Yanji city from 2018 to 2024.

All six streets and four towns in Yanji reported varicella cases from 2018 to 2024, with annual incidence rates ranging from 7.29 per 100,000 population (Xinxing Street) to 20.16 per 100,000 population (Jin Xue Street). Although not statistically significant, the incidence rate decreased in all areas (Figure 4).

### 3.3. Population Distribution

#### 3.3.1. Sex Distribution

**Figure 5:**
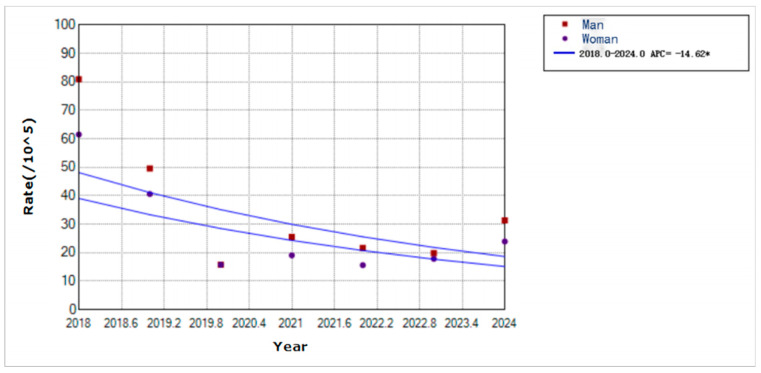
Trends in varicella incidence rates by sex in Yanji city from 2018 to 2024. * Indicates that the Annual Percent Change (APC) is significantly different from zero at the alpha = 0.05 level.

From 2018 to 2024, 1366 male and 1086 female cases were reported, with a sex ratio of 1.26:1. Additionally, the annual incidence rates were 34.93 per 100,000 males and 27.77 per 100,000 females. Moreover, varicella incidence declined for both sexes, with a faster decline in males (APC = −14.62, *p* < 0.01) than in females (Figure 5).

#### 3.3.2. Age Distribution

**Figure 6:**
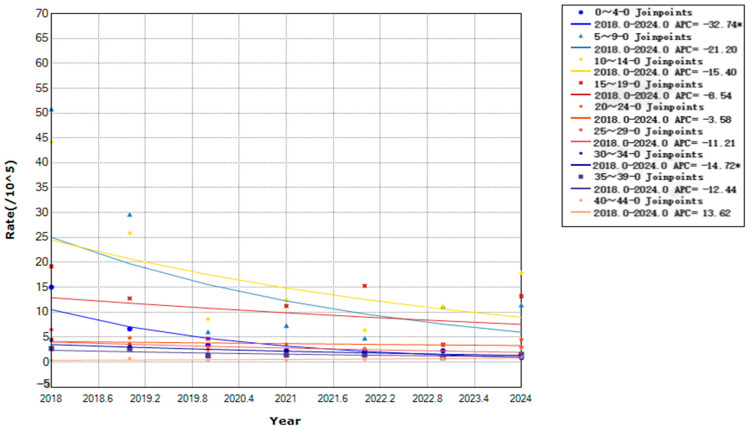
Trends in varicella incidence rates by age group in Yanji city from 2018 to 2024. * Indicates that the Annual Percent Change (APC) is significantly different from zero at the alpha = 0.05 level.

From 2018 to 2024, children aged 0–12 years accounted for 1337 cases (54.53%), whereas those aged 35–49 years accounted for 96 cases (3.92%). Notably, the annual incidence rates per 100,000 population by age group were: 0–4 years (4.67), 5–9 years (17.32), 10–14 years (18.08), 15–19 years (11.41), 20–24 years (3.80), 25–29 years (3.12), 30–34 years (2.38), 35–39 years (1.71), and ≥40 years (0.89). Additionally, the 10–14 years age group had the highest incidence, with a declining trend (APC = −8.79%, *p* < 0.01), followed by the 5–9 years age group. Moreover, the 0–4 age years group showed the fastest decline (APC = −32.74%, *p* < 0.01), followed by the 30–34 years age group (APC = −14.72%, *p* < 0.01). However, the other age groups showed no significant trends (Figure 6).

#### 3.3.3. Occupational Distribution

**Figure 7:**
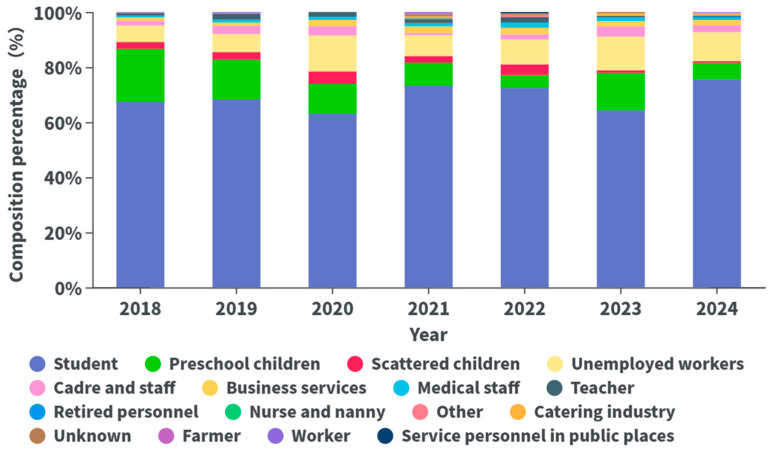
Occupational distribution of varicella cases in Yanji city from 2018 to 2024.

In this study, the top three occupations among varicella cases from 2018 to 2024 were students (69.86%), preschool children (13.34%), and unemployed/household workers (8.24%) (Figure 7).

#### 3.3.4. Vaccination Status

In total, 31,088 doses of varicella vaccine were administered in Yanji from 2018 to 2020, including 5091 doses of the Changchun Baike vaccine (16.38% of total doses). In 2018, 3491 doses of the Changchun Baike vaccine were administered (28.3% of total vaccinations), with Henan street having the highest proportion (35.7%) and Sandaowan town the lowest (0%). In 2019, 641 doses were administered (7.34%), with Jin Xue street having the highest proportion (11.3%) and Sandaowan town the lowest (0%). In 2020, 959 doses were administered (9.58%), with Chaoyangchuan and Sandaowan towns having the highest (76.1%) and lowest (0%) proportions, respectively (Table 1).

### 3.4. Vaccine Protective Efficacy

Significant age differences existed between vaccinated and unvaccinated groups before matching in the <15 years and ≥15 years age groups across all observation periods (*p* < 0.001). After 1:1 propensity score matching (PSM), no significant age differences remained (*p* > 0.05; Supplementary Tables S2–S4).

Cox regression showed consistently high protective efficacy of Changchun Baike varicella vaccine across the three periods (Table 2). In the 2018–2022 period, the one-dose, two-dose, and one- or two-dose schedules all demonstrated an adjusted vaccine effectiveness (aVE) of 99.0% (95% CI: 98.0–99.0%). In the 2019–2023 period, the one-dose and two-dose schedules yielded an aVE of 99.0% (95% CI: 97.0–100.0%), and the one- or two-dose schedule also achieved 99.0% (95% CI: 97.0–100.0%). In the 2020–2024 period, the one-dose schedule provided an aVE of 98.0% (95% CI: 97.0–99.0%), while the two-dose and one- or two-dose schedules both showed 99.0% (95% CI: 98.0–100.0%). The risk of varicella in vaccinated groups was 0.01–0.02 times that of the unvaccinated reference group (all *p* < 0.001). The two-dose schedule demonstrated superior protection against breakthrough infections compared with the single-dose schedule.

## 4. Discussion

In this study, we investigated the epidemiological characteristics of varicella and the protective efficacy of Changchun Baike in Yanji from 2018 to 2024. In total, 2452 cases were reported from 2018 to 2024, with an average annual incidence rate of 62.71 per 100,000 population. Notably, the incidence rate declined from 142.37 per 100,000 in 2018 to 55.25 per 100,000 population by 2024. This decline is closely linked to the widespread use of the Changchun Baike varicella vaccine, which reduces the risk of VZV transmission through humoral and cellular immunity [7]. Additionally, cases were concentrated among preschool and school-aged children, with a slightly higher incidence in males than in females. Moreover, the decline in the incidence rate was faster in males than in females during the period under consideration. Among the areas surveyed, the densely populated Jin Xue and Beishan streets had relatively high incidence rates. Additionally, the economically underdeveloped Sandaowan town had a higher transmission risk due to low vaccination coverage [9,10]. Vaccine efficacy analysis showed an adjusted efficacy of 98.0–99.0% (95% CI: 97.0–100.0%) for the one-dose schedule and 99.0% (95% CI: 98.0–100.0%) for the two-dose schedule, with latter schedule significantly outperforming the former schedule in preventing breakthrough infections [11,12]. However, breakthrough infections and inadequate vaccination coverage suggest the need for optimized vaccination strategies to further reduce the disease burden [13,14].

Consistent with findings in Qingyang, Gansu, and Shenyang [2,15], varicella incidence exhibited a distinct seasonal pattern, with peaks from April to July (31.83%) and October to January (51.71%). Winter peaks are associated with increased indoor gatherings and higher respiratory droplet transmission risk in cold conditions, whereas late spring to early summer peaks are correlated with increased school-related contact and social activities [7]. Despite the declining incidence, the seasonal pattern remained unchanged, indicating that VZV transmission is driven by climate and behavioral factors [16]. Enhanced surveillance in schools and childcare facilities during peak seasons is crucial for identifying and controlling outbreaks.

Spatially, all six streets and four towns reported cases with significant variations in incidence rates. Jin Xue and Beishan streets, with high population density and frequent mobility, were high-risk areas, reflecting the role of social contact in transmission chains [17,18,19]. Economically disadvantaged areas, such as Sandaowan town, had a higher transmission risk due to poor sanitation and low vaccination coverage [20]. Targeted interventions, including increased vaccination coverage, improved public health infrastructure, and health education, are necessary to reduce transmission in high-risk areas.

Regarding population distribution, cases were concentrated among children aged 0–14 years, with the 10–14 years age group having the highest incidence, followed by the 5–9 years age group. Preschool- and school-aged children are more susceptible to the disease than adults because of their underdeveloped immune systems and frequent contact in crowded settings [21,22]. Notably, the incidence rate increased in the ≥40 years age group, possibly due to waning single-dose vaccine efficacy or lack of vaccination in some adults [23]. Additionally, the higher incidence rate in males than in females may be due to more active social interactions, poorer hygiene, and lower parental acceptance of vaccination among males [24,25,26]. Male patients are more prone to complications, such as skin infections and pneumonia, which may increase the healthcare burden [27]. Moreover, occupational distribution highlighted students, preschool children, and unemployed/household workers as primary case groups, driven by school and childcare environments, low vaccination coverage, and limited parental awareness of vaccine safety [28,29].

Importantly, the Changchun Baike varicella vaccine demonstrated excellent protective efficacy. One-dose aVE was 99.0% (95% CI: 98.0–99.0%) and two-dose was 99.0% (95% CI: 98.0–99.0%) from 2018 to 2022; both were 99.0% (95% CI: 97.0–100.0%) from 2019 to 2023; and one-dose was 98.0% (95% CI: 97.0–99.0%) and two-dose was 99.0% (95% CI: 98.0–100.0%) from 2020 to 2024. Cox regression confirmed that two-dose vaccination had a lower hazard ratio, offering significant advantages in preventing breakthrough infections [30]. Nevertheless, the observed high adjusted vaccine effectiveness (98–99%) is notably higher than many international real-world estimates for single-dose (typically 80–90%) and two-dose (90–98%) varicella vaccination schedules [31]. Several factors may contribute to this finding, including potential under-reporting of mild breakthrough cases among vaccinated individuals, healthier vaccinator bias, residual confounding despite propensity score matching, and high specificity of case confirmation (exclusion of suspected cases). Additionally, non-pharmaceutical interventions implemented during the COVID-19 pandemic (e.g., school closures, mask-wearing, and reduced social mixing) likely contributed independently to the marked decline in varicella incidence observed from 2020 onward [32,33]. Although the temporal association with increasing vaccination coverage supports a substantial vaccine impact, these co-interventions preclude attribution of the entire reduction to vaccination alone. Additionally, the incidence of breakthrough infections suggests that vaccine efficacy is influenced by individual immune responses, age at vaccination, and storage conditions [34]. Children vaccinated at ≤15 months had a higher infection risk, and single-dose antibody titers wane over time, whereas two doses significantly extend protection [30]. Economic barriers and insufficient health education limit second-dose uptake [16]. For example, the free two-dose policies in Shanghai and Tianjin significantly reduced the incidence of the disease [35,36]. Additionally, schools and childcare facilities should implement vaccination verification and routine VZV surveillance to curb varicella outbreaks [37]. Future research should focus on the long-term efficacy of two-dose vaccination schedule and the pathogenesis of breakthrough infections to optimize strategies and strengthen control measures.

### Study Limitations

This study has several important limitations. First, as an observational study relying on routine surveillance data, laboratory confirmation was not routinely performed for all cases, potentially leading to case misclassification. Second, despite the use of propensity score matching, residual confounding by unmeasured factors (e.g., socioeconomic status, geographic factors, and healthcare-seeking behavior) cannot be entirely excluded. Third, funding was provided by the vaccine manufacturer, raising concerns about potential bias; although data were sourced from an independent platform and analyses followed standardized protocols, the possibility of influence cannot be fully excluded. Finally, underreporting of mild cases in vaccinated children is possible within passive surveillance systems.

## Figures and Tables

**Table 1:** Proportion of Changchun Baike varicella vaccine in Yanji City and its regions, 2018–2020.

Year	Region	Total Varicella Vaccinations	Changchun Baike Vaccinations	Proportion (%)
2018	Yanji city	12,342	3491	28.3
	Henan street	1780	636	35.7
	Sandaowan town	0	0	0
2019	Yanji city	8735	641	7.34
	Jin Xue street	1203	137	11.3
	Sandaowan town	0	0	0
2020	Yanji city	10,011	959	9.58
	Chaoyangchuan town	210	160	76.1
	Sandaowan town	0	0	0
2018–2020	Yanji city	31,088	5091	16.38

Note: Proportions were calculated as (Changchun Baike vaccinations/Total varicella vaccinations) × 100. Only the city-wide total and the regions with the highest and lowest proportions each year are shown for brevity; full regional data are available in Supplementary Table S1.

**Table 2:** Cox regression analysis of Changchun Baike Varicella vaccine, 2018–2024.

Year	Variable	β	S.E	Z	*p*	HR (95% CI)	aVE (%) (95% CI)
2018–2022	Doses						
	0	-	-	-	-	1.00 (Reference)	-
	1	−4.68	0.29	−16.14	<0.001	0.01 (0.01–0.02)	99.0 (98.0–99.0)
	2	−4.70	0.30	−15.51	<0.001	0.01 (0.01–0.02)	99.0 (98.0–99.0)
	All Doses	−4.22	0.21	−20.09	<0.001	0.01 (0.01–0.02)	99.0 (98.0–99.0)
2019–2023	Doses						
	0	-	-	-	-	1.00 (Reference)	-
	1	−4.99	0.71	−7.04	<0.001	0.01 (0.00–0.03)	99.0 (97.0–100.0)
	2	−4.99	0.71	−7.04	<0.001	0.01 (0.00–0.03)	99.0 (97.0–100.0)
	All Doses	−4.59	0.58	−7.91	<0.001	0.01 (0.01–0.02)	99.0 (97.0–100.0)
2020–2024	Doses						
	0	-	-	-	-	1.00 (Reference)	-
	1	−3.82	0.58	−6.54	<0.001	0.02 (0.00–0.07)	98.0 (97.0–99.0)
	2	−5.23	0.58	−9.04	<0.001	0.01 (0.00–0.02)	99.0 (98.0–100.0)
	All Doses	−4.63	0.41	−10.65	<0.001	0.01 (0.01–0.03)	99.0 (98.0–100.0)

Abbreviations: β, regression coefficient; S.E, standard error; Z, z-statistic; *p*, *p*-value; HR, hazard ratio; aVE, adjusted vaccine effectiveness; CI, confidence interval; -, not applicable. Estimates were derived from Cox proportional hazards models after 1:1 propensity score matching (nearest-neighbor, caliper = 0.2). Detailed covariate balance and sample size distributions before and after matching are provided in Supplementary Tables S2–S4.

## Data Availability

The datasets presented in this article are not readily available to avoid misuse of data and information. The datasets used or analyzed during the current study are available from the corresponding author, Yihua Li, on reasonable request. Requests to access the datasets should be directed to Yihua Li, liyihua@ybu.edu.cn.

## Supplementary Materials

The following supporting information can be downloaded at: https://www.mdpi.com/article/10.3390/vaccines14010042/s1, Table S1: Proportion of Changchun Baike varicella vaccine in Yanji City and its regions, 2018–2020. Table S2: Distribution characteristics of vaccinated and unvaccinated groups before and after PSM, 2018–2022. Table S3: Distribution characteristics of vaccinated and unvaccinated groups before and after PSM, 2019–2023. Table S4: Distribution characteristics of vaccinated and unvaccinated groups before and after PSM, 2020–2024.

## References

[1] Rasizadeh R., Shamekh A., Shiri Aghbash P., Bannazadeh Baghi H. (2023). Comparison of human monkeypox, chickenpox and smallpox: A comprehensive review of pathology and dermatological manifestations. Curr. Med. Res. Opin..

[2] Bai S. (2020). Epidemiological characteristics of varicella among primary and secondary school students in Shenyang City, 2006–2018. Chin. J. Sch. Health.

[3] Ayoade F., Kumar S. (2022). Varicella-Zoster Virus (Chickenpox). StatPearls [Internet].

[4] (2023). Overview of National Notifiable Infectious Diseases in 2022 [EB/OL].

[5] Dong P.M., Wang M., Liu Y.M. (2020). Epidemiological characteristics of varicella in China, 2016–2019. Chin. J. Vaccines Immun..

[6] Wang H.Y., Liu F. (2021). Progress in research on epidemiological characteristics and vaccine protective efficacy of varicella in China. Prev. Med. Forum.

[7] Di Pietrantonj C., Rivetti A., Marchione P., Debalini M.G., Demicheli V. (2021). Vaccines for measles, mumps, rubella, and varicella in children. Cochrane Database Syst. Rev..

[8] Pan X., Shu M., Ma R., Fang T., Dong H., Sun Y., Xu G. (2021). Varicella breakthrough infection and effectiveness of 2-dose varicella vaccine in China. Vaccine.

[9] Lin M., Yang T., Deng P., Yang L., Xue C. (2025). Analysis on the Epidemiological Characteristics of Breakthrough Varicella Cases and Incremental Effectiveness of 2-Dose Varicella Vaccine in China. Vaccines.

[10] Li W., Liu L.L., Tan H.L., Zhuang C.Y., Zeng Y., Zhu Y.F., Ye B.L., Wei H.M., Li G. (2025). Protective efficacy evaluation of trivalent inactivated influenza vaccine among primary and secondary school students in Longgang District, Shenzhen, during the 2023–2024 influenza season. Chin. J. Vaccines Immun..

[11] Zhang R.Y., Jiang Z., Gao L. (2023). Epidemiological characteristics of varicella in Guiyang City, 2010–2022. Mod. Prev. Med..

[12] Luo R.J., Wen Y., Cheng Y.P., Chen N.X., Huang F., Chen Z.G., Zhang Z., Lü Q.Y. (2024). Epidemiological trends of major respiratory infectious diseases among people aged 6–19 years in Shenzhen, 2013–2022. Chin. J. Trop. Med..

[13] Zhang X.Q., Lü Y., Wang Y., Pan F., Chen Y.F., Zhang H., Yang H., Shao M.H., Cheng K., Qin W. (2023). Epidemiological characteristics and vaccine protective efficacy of varicella in Liu’an City, 2010–2022. Chin. J. Vaccines Immun..

[14] Lin M.Z., Ding M.H., Deng P.F., Wang Q.Z., Fei Y., Xue C.Y. (2022). Epidemiological characteristics of varicella in Pudong New Area, Shanghai, 2011–2020. Chin. J. Biol..

[15] Ding X., Ren D.F., Gao Q.R., Ning L.T., Xiao Y.F. (2021). Epidemiological characteristics of varicella in Tongren City, 2010–2020. Mod. Prev. Med..

[16] Chen Q., Wang M.C., Zeng X.P. (2021). Epidemiological characteristics of infectious diseases in schools and childcare facilities in Haikou City, 2015–2019. Mod. Prev. Med..

[17] Wu J.J., Zou L.P., He Y.J., Guo L., Xie Y.Z., Han Y., Wang Q.F. (2024). Epidemiological characteristics and vaccine protective efficacy of varicella in Jinan City, 2006–2022. Mod. Prev. Med..

[18] Wang Z., Chen L., Lu F., Peng J., Huang F., Xie X., Kong D. (2024). Analysis of the implementation effect and evaluation of the vaccine protection effect of the live attenuated varicella vaccine program for school-age children in Bao’an district of Shenzhen, China. Hum. Vaccin. Immunother..

[19] Zhang Z., Zhang Y., Yu J., Dong C., Zhang J., Liu N., Qian C., Luan L. (2023). Seroprevalence rates in children aged 3–6 years after implementing a two-dose varicella vaccination: A observational study. Hum. Vaccin. Immunother..

[20] Li Y., Xu F., Liu M., Teng S., Liang F., Wang F. (2024). Effectiveness of two-dose vs. one-dose varicella vaccine in children in Shanghai, China: A prospective cohort study. Front. Public. Health.

[21] Wang L., Wang M.M., Xu C.D., Wang P.H., You M.Y., Li Z.H., Chen X.M., Liu X.Y., Li X.D., Wang Y.Y. (2024). Spatial Dynamics of Chickenpox Outbreaks in Rapidly Developing Regions: Implications for Global Public Health. Biomed. Environ. Sci..

[22] Williame I., George M., Shah H.A., Homer N., Alderson D., Jamet N. (2023). Healthcare resource use and costs of varicella and its complications: A systematic literature review. Hum. Vaccin. Immunother..

[23] Zeng T., Lian C.-X., Zhang X.-Y., Liu P.-Q., Ao J., Zhou G.-F., Chen X.-D., Huang D.-D., Hu D.-G., Chen X. (2025). Clinical symptoms and molecular epidemiologic characteristics of varicella patients among children and adults in Ganzhou, China. Virol. J..

[24] Luan G., Yao H., Yin D., Liu J. (2024). Trends and Age-Period-Cohort Effect on Incidence of Varicella Under Age 35—China, 2005–2021. China CDC Wkly..

[25] Zhang M., Gui G.-P., Guo F., Fan X.-F., Zha R.-S. (2020). A Centralized Outbreak of Varicella among Children Attending Preschool in Suzhou, China. Biomed. Res. Int..

[26] Ribeiro M.Z., Kupek E., Ribeiro P.V.Z., Pinheiro C.E.A. (2020). Impact of the tetra viral vaccine introduction on varicella morbidity and mortality in the Brazilian macro regions. J. Pediatr..

[27] Hu Y.H., Luo X.F., Lyu M., Yin D.P. (2021). A Meta-analysis on varicella-zoster virus antibody levels in healthy population in China. Zhonghua Liu Xing Bing Xue Za Zhi.

[28] Liu X.Y., Wang M.M., You M.Y., Wang P.H., Wang T.Q., Chen X.M., Xu C.D., Li X.D., Wang L., Hu Y.H. (2024). Epidemiological characteristics and influencing factors of public health emergency events of varicella in the Beijing-Tianjin-Hebei region, 2006–2021. Zhonghua Yu Fang Yi Xue Za Zhi.

[29] Tam W.W., Chan J., Lo K.K., Lee A., Chan P.K., Chan D., Nelson E.A.S. (2015). Parental Attitudes and Factors Associated With Varicella Vaccination in Preschool and Schoolchildren in Hong Kong: A Cross-Sectional Study. Medicine.

[30] Zhu H., Zhao H., Ou R., Zeng Q., Hu L., Qiu H., Sharma M., Ye M. (2020). Spatiotemporal Epidemiology of Varicella in Chongqing, China, 2014–2018. Int. J. Environ. Res. Public Health.

[31] Liang H., Qi X., Chen Y., Pan X. (2025). Surveillance of Adverse Events Following Varicella Vaccine Immunization in Zhejiang Province, China, from 2020 to 2022. Vaccines.

[32] Sabale U., Jarmale L., Murtagh J., Pawaskar M., Bencina G. (2023). Impact assessment of immunization and the COVID-19 pandemic on varicella across Europe using digital epidemiology methods: A descriptive study. PLoS ONE.

[33] Wu Q., Liu Y., Xia J., Wu T., Wang D., Lu J. (2020). The impact of COVID-19 control measures on the morbidity of varicella, herpes zoster, rubella and measles in Guangzhou, China. Immun. Inflamm. Dis..

[34] Chen Y.-F., Zhou Q., Liu J.-Y., Gong R.-J., Mao S.-Q., Ye Z.-J., Wu Q.-S. (2020). Characteristics of within-household varicella transmission events associated with school outbreaks in Shanghai, China, 2009–2018. Epidemiol. Infect.

[35] Chen D., Li Y., Wu Q. (2021). Effectiveness of varicella vaccine as post-exposure prophylaxis: A meta-analysis. Hum. Vaccin. Immunother..

[36] Varela F.H., Pinto L.A., Scotta M.C. (2019). Global impact of varicella vaccination programs. Hum. Vaccines Immunother..

[37] Chemaitelly H., AlMukdad S., Ayoub H.H., Altarawneh H.N., Coyle P., Tang P., Yassine H.M., Al-Khatib H.A., Smatti M.K., Hasan M.R. (2022). COVID-19 Vaccine Protection among Children and Adolescents in Qatar. N. Engl. J. Med..

