# Supplementary material for: High Effectiveness of the Changchun Baike Varicella Vaccine in a Real-World Outbreak Setting: An Observational Study from Yanji City, China
Source: Vaccines (Basel). 2025 Dec 30;14(1):42. doi: 10.3390/vaccines14010042 (PMC12846119; doi:10.3390/vaccines14010042)
Supplement: Supplementary file 1 [file vaccines-14-00042-s001.zip › vaccines-4035474-supplementary.pdf]

Table S1. Proportion of Changchun Baike varicella vaccine in Yanji City and its regions, 2018–2020.

Region	2018 Total varicella vaccinations	2018 Changchun Baike vaccinations	2018 Proportion (%)	2019Total varicella vaccinations	2019 Changchun Baike vaccinations	2019 Proportion (%)	2020Total varicella vaccinations	2020 Changchun Baike vaccinations	2020 Proportion (%)
Yanji city	12,342	3,491	28.3	8735	641	7.34	10011	959	9.58
Jin Xue street	2,198	631	28.7	1203	137	11.3	1249	85	6.8
Beishan street	1,999	487	24.4	1169	39	3.3	1256	43	3.4
Xinxing street	1,771	373	21.1	766	57	7.4	996	40	4
Jiangong street	2,647	723	27.3	2120	161	7.6	1917	269	14
Henan street	1,780	636	35.7	1619	161	9.9	1171	15	1.2
Gongyuan street	2,062	522	25.3	1602	202	10.1	2105	232	11
Chaoyangchuan town	234	71	30.3	164	15	9.1	210	160	76.1
Yilan town	523	25	4.7	25	5	20	20	10	50
Xiaoying town	77	23	29.8	69	2	2.8	139	105	75.5
Sandaowan town	0	0	0	0	0	0	0	0	0

Note：Proportions were calculated as (Changchun Baike vaccinations / Total varicella vaccinations) × 100.

Table S2. Distribution characteristics of vaccinated and unvaccinated groups before and after PSM, 2018–2022.

	**Before matching**						**After matching**					
Variable	Unvaccinated group		Vaccinated group		X2	p	Unvaccinated group		Vaccinated group		X2	p
	Cases	Proportion (%)	Cases	Proportion (%)			Cases	Proportion (%)	Cases	Proportion (%)		
1 Dose												
< 15	70882	12.74	1904	98.81	12543.498	<.001	1904	98.81	1904	98.81	0.000	1.000
≥ 15	485363	87.26	23	1.19			23	1.19	23	1.19		
2 Doses												
< 15	71241	12.80	1559	99.43	10345.675	<.001	1559	99.43	1559	99.43	0.000	1.000
≥ 15	485363	87.20	9	0.57			9	0.57	9	0.57		
1 or 2 Doses												
< 15	69386	12.55	3463	99.08	19121.808	<.001	3463	99.08	3463	99.08	0.000	1.000
≥ 15	485091	87.45	32	0.92			32	0.92	32	0.92		

Note: After matching, 1,927 pairs for one-dose, 1,568 pairs for two-dose, and 3,495 pairs for one- or two-dose analyses were included.

Table S3. Distribution characteristics of vaccinated and unvaccinated groups before and after PSM, 2019–2023.

	**Before matching**						**After matching**					
Variable	Unvaccinated group		Vaccinated group		X2	p	Unvaccinated group		Vaccinated group		X2	p
	Cases	Proportion (%)	Cases	Proportion (%)			Cases	Proportion (%)	Cases	Proportion (%)		
1 Dose												
< 15	72273	12.90	321	93.59	1660.372	<.001	321	93.59	321	93.59	0.000	1.000
≥ 15	488127	87.10	22	6.41			22	6.41	22	6.41		
2 Doses												
< 15	72273	12.90	321	93.59	1660.372	<.001	321	93.59	321	93.59	0.000	1.000
≥ 15	488127	87.10	22	6.41			22	6.41	22	6.41		
1 or 2 Doses												
< 15	72139	12.79	642	93.59	6371.477	<.001	642	93.59	642	93.59	0.000	1.000
≥ 15	488677	87.21	44	6.41			44	6.41	44	6.41		

Note: After matching, 343 pairs were included for one-dose analysis, 343 pairs for two-dose analysis, and 686 pairs for one- or two-dose analysis.

Table S4. Distribution characteristics of vaccinated and unvaccinated groups before and after PSM, 2020–2024.

	**Before matching**						**After matching**					
Variable	Unvaccinated group		Vaccinated group		X2	p	Unvaccinated group		Vaccinated group		X2	p
	Cases	Proportion (%)	Cases	Proportion (%)			Cases	Proportion (%)	Cases	Proportion (%)		
1 Dose												
< 15	72273	12.90	325	93.66	1660.372	<.001	321	93.59	321	93.59	0.000	1.000
≥ 15	488127	87.10	22	6.34			22	6.41	22	6.41		
2 Doses												
< 15	72130	12.85	606	99.02	3686.416	<.001	321	93.59	321	93.59	0.000	1.000
≥ 15	489084	87.15	6	0.97			22	6.41	22	6.41		
1 or 2 Doses												
< 15	71857	12.79	931	97.08	6371.477	<.001	931	97.08	931	97.08	0.000	1.000
≥ 15	489062	87.21	28	2.92			28	2.92	28	2.92		

Note:After matching, 343 pairs were included for one-dose analysis, 343 pairs for two-dose analysis, and 959 pairs for one- or two-dose analysis.
